# Bacteria coated cathodes as an *in-situ* hydrogen evolving platform for microbial electrosynthesis

**DOI:** 10.1038/s41598-020-76694-y

**Published:** 2020-11-16

**Authors:** Elisabet Perona-Vico, Laura Feliu-Paradeda, Sebastià Puig, Lluis Bañeras

**Affiliations:** 1 Molecular Microbial Ecology Group, Institute of Aquatic Ecology, University of Girona, Maria Aurèlia Capmany 40, 17003 Girona, Spain; 2 LEQUiA, Institute of the Environment, University of Girona, Maria Aurèlia Capmany 69, 17003 Girona, Spain

**Keywords:** Applied microbiology, Biotechnology

## Abstract

Hydrogen is a key intermediate element in microbial electrosynthesis as a mediator of the reduction of carbon dioxide (CO_2_) into added value compounds. In the present work we aimed at studying the biological production of hydrogen in biocathodes operated at − 1.0 V *vs.* Ag/AgCl, using a highly comparable technology and CO_2_ as carbon feedstock. Ten bacterial strains were chosen from genera *Rhodobacter*, *Rhodopseudomonas*, *Rhodocyclus*, *Desulfovibrio* and *Sporomusa*, all described as hydrogen producing candidates. Monospecific biofilms were formed on carbon cloth cathodes and hydrogen evolution was constantly monitored using a microsensor. Eight over ten bacteria strains showed electroactivity and H_2_ production rates increased significantly (two to eightfold) compared to abiotic conditions for two of them (*Desulfovibrio paquesii* and *Desulfovibrio desulfuricans*). *D. paquesii* DSM 16681 exhibited the highest production rate (45.6 ± 18.8 µM min^−1^) compared to abiotic conditions (5.5 ± 0.6 µM min^−1^), although specific production rates (per 16S rRNA copy) were similar to those obtained for other strains. This study demonstrated that many microorganisms are suspected to participate in net hydrogen production but inherent differences among strains do occur, which are relevant for future developments of resilient biofilm coated cathodes as a stable hydrogen production platform in microbial electrosynthesis.

## Introduction

Microbial electrosynthesis (MES) is engineered to use electric power and carbon dioxide (CO_2_) as the only energy and carbon sources in reductive bioelectrochemical processes for biosynthesis^1^. Among potential uses of MES, alternative biofuels production copes for most of the scientific attention and deserves an intense research activity^2,3^. Some microorganisms are able to transfer electrons to or from a poised solid electrode^4^, a talent that contributed to develop a broad range of practical applications from bioenergy to water treatment^5,6^. Bioelectrochemical systems (BES) exploit the capacity of these electroactive microorganisms able to capture electrons and transform them into soluble energy containing compounds (organic multicarbon molecules)^7^. A major limitation in BES processes is the rate at which microorganisms acquire electrons from solid state electrodes for CO_2_ reduction^8^. Several studies have proposed hydrogen (H_2_) as the principal electron donor intermediary in the production of commodity chemicals from carbon dioxide and electricity^9–11^. Molecules such as H_2_, carbon monoxide (CO) and formate are the most preferable for microbial catalysts^12^. Microbial electrosynthesis will be reinforced by the integration of proper H_2_-producing microbial catalysts.

Metabolically, surplus hydrogen production for most anaerobic microorganisms is an induced response in order to avoid accumulation of reduced cofactors (NAD, NADP, FAD, ferredoxins, and others) so that metabolic processes can continue^13^. Hydrogenases and nitrogenases are among the most widespread enzymes involved in proton reduction for hydrogen production. Hydrogenases are responsible of the reversible reaction to convert protons and electrons into hydrogen (2H^+^  + 2e^−^ $\leftrightarrow$ H_2_). Contrarily, nitrogenases naturally produce H_2_ as a by-product of nitrogen fixation (N_2_ + 8e^−^  + 8H^+^  + 16ATP $\leftrightarrow$ 2NH_3_ + H_2_ + 16ADP + 16 P_i_). Under nitrogen limitation, nitrogenases function as a hydrogenase and only produce H_2_ by proton reduction to molecular hydrogen (2H^+^  + 2e^−^  + 4ATP $\leftrightarrow$ H_2_ + 4ADP + 4P_i_)^13,14^.

In biocathodes, H_2_ is produced either abiotically (pure electrocatalytic process) or biotically, with the participation of living microorganisms or isolated enzymes. Abiotic or electrocatalytic H_2_ is produced when using carbon-based materials (i.e. graphite cathodes) at cathode potentials below − 0.8 V *vs*. Ag/AgCl^9^. Biologically produced H_2_ (BioH_2_) have been proven in biocathodes by using both pure and mixed microbial cultures. *Geobacter sulfurreducens*, *Rhodobacter capsulatus*, and *Desulfovibrio* spp. catalyze hydrogen production at cathode potentials below − 0.8 V *vs.* Ag/AgCl^10,15–17^. Microbial community characterizations demonstrated that highly H_2_ producing biocathodes were enriched mainly by *Proteobacteria*^10,11,18^. Croese and co-workers described a cathodic microbial community mainly composed of *Deltaproteobacteria* in which *Desulfovibrio* spp. were the most abundant^18^. *Alpha-* and *Betaproteobacteria* (*Rhodocyclaceae*) have also been highlighted to be mediating H_2_ production in cathodes^10,11^. In addition, increased H_2_ production rates in the presence of cell-free exhausted medium from cultures of *Sporomusa sphaerodies*, *Sporomusa ovata* and *Methanococcus maripaludis* have also been confirmed^19–21^. The authors demonstrated that the former presence of microorganisms in the reactor had changed the electrode surface via metal deposition (nickel and cobalt) leading to an increased H_2_ production^19^. Alternatively, free enzymes (hydrogenases and formate dehydrogenases) previously released by microorganisms^20,21^ could also be deposited in the electrode surface reinforcing H_2_ production yields.

Another feasible strategy aiming to improve H_2_ production in biocathodes is the integration of low-cost metal-based cathode materials such as cobalt phosphide, molybdenum disulfide and nickel-molybdenum with putatively electroactive microorganisms. Although its integration with the required conditions for microbial growth might cause toxicity towards microorganisms^22^, some of these materials have been demonstrated as a promising and biocompatible electrocatalytic H_2_-producing platform, while combining with CO_2_-reducing and H_2_-utilizing bacteria (like *S. ovata* and *M. maripaludis*) ensuring higher value-added chemicals production^23^.

In the present work we aimed at assessing the capacity of ten strains of *Rhodobacter*, *Rhodopseudomonas*, *Rhodocyclus*, *Desulfovibrio* and *Sporomusa* previously reported as potential cathodic H_2_ producers. This approach may allow selecting potential candidates to stablish resilient co-cultures, ideally composed of a H_2_ producing strain in combination with a homoacetogen, for microbial electrosynthesis processes and electro-fermentation. We used an optimized experimental protocol which could facilitate the comparison among strains thus limiting the effect of heterogenous reactor designs and analytical tools which are found in the literature. H_2_ evolution was continuously monitored by means of an H_2_ microsensor placed directly in contact with liquid medium and close to cathode surface. H_2_ production efficiencies in monospecific biofilms of each strain were analyzed repeatedly and compared to abiotic conditions.

## Results and discussion

### Electrocatalytic H_2_ production at carbon cloth electrodes

Accurate choice of control conditions in reactor set-ups (as abiotic controls) is mandatory to avoid data deviation and facilitate interpretation^20^. Several tests were carried out to determine the electrocatalytic (or abiotic) H_2_ production in carbon cloth electrodes. The use of a fixed methodology and an exhaustive analysis of control experiments facilitated comparison among strains and detection of relevant biotic effects. Carbon cloth electrodes were operated at different potentials (− 0.6, − 0.8 and − 1.0 V *vs.* Ag/AgCl) and H_2_ evolution was monitored. Under these conditions, catalytic H_2_ was only detected when cathodes were poised at − 0.8 and − 1.0 V *vs.* Ag/AgCl (Supplementary Table S1). Independently of the medium used, higher H_2_ production rates (8.4 ± 3.0, 6.4 ± 1.8 and 5.1 ± 0.8 µM min^−1^, respectively) were achieved at − 1.0 V.

Electrocatalytic H_2_ production is conditioned by several factors, such as liquid medium composition, temperature, overpressure, electrode material, reactor designs and/or operating modes^22–25^. To estimate if medium composition was affecting hydrogen production, ionic losses were calculated (Supplementary Table S2). DSM 311 and Aulenta et al*.* modified media had similar ionic losses (+ 85 and + 89 mV, respectively). Modified DSM 27 had slightly higher ionic loss (+ 184 mV) compared to the other media. These differences were related to the different salinities, however observed differences should not have a significant effect on catalytic hydrogen productions.

Despite special care was applied to minimize effects in cathode sizes and qualities, differences in H_2_ production rates were detected between carbon cloth electrodes, suggesting that parameters such as material integrity or the presence of impurities, could be affecting H_2_ production. Consequently, it was necessary to measure abiotic H_2_ concentrations for each carbon cloth electrode later used for monospecific biofilm formation to ensure proper results interpretation.

### Formation of monospecific biofilms and stability

Abundance of the 16S rRNA gene was used as a proxy for estimating bacterial density in BES. Although 16S rRNA gene copies could be translated into cell abundance in view of 16S rRNA copies per unit genome^26^, no such transformation was performed since differences were only analyzed in terms of biofilm stability of individual strains during experiments. As expected, the presence of bacteria from the beginning of the BES operation was confirmed. 16S rRNA gene copies ranged from 3.7 × 10^6^ to 4.5 × 10^5^ gene copies/cm^2^ in biofilm samples, and from 1.5 × 10^8^ to 9.7 × 10^4^ gene copies/mL in bulk liquid.

**Figure 1:**
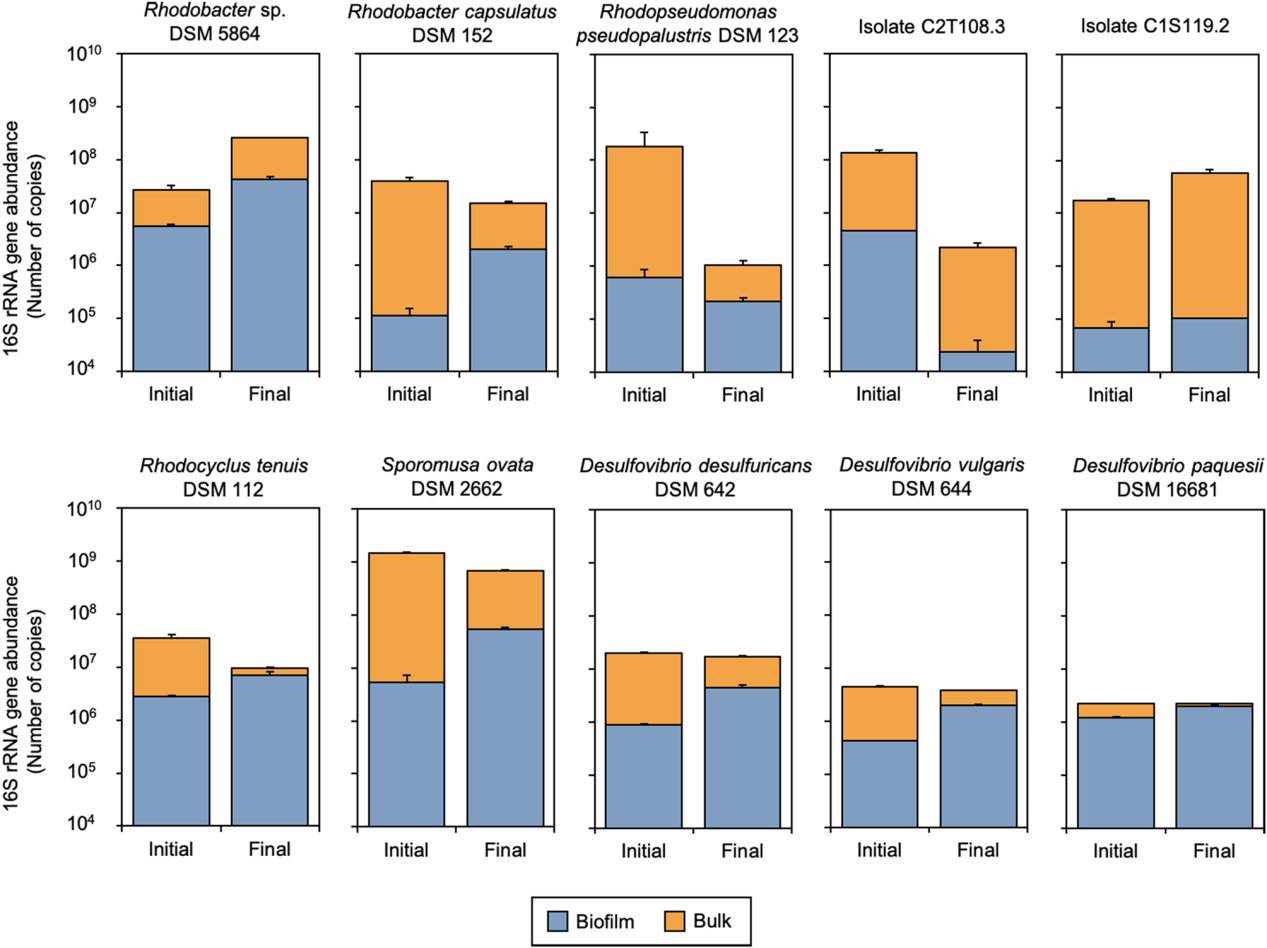
Abundance of 16S rRNA gene into biofilm and bulk samples. Logarithmic scale is used to show number of copies from samples taken at the beginning (Initial) and at the end of BES operation (Final).

Proportions between abundances measured before and after BES operation were used to assess the short-term stability of the biofilm. Except for *Rhodopseudomonas pseudopalustris* DSM 123 and isolate C2T108.3, all bacterial strains tended to remain attached into the cathode during the operation, or even grow as a biofilm, since an increase in gene copies concentrations was found (more than tenfold increase for *Rhodobacter capsulatus* DSM 152; Fig. 1). Total 16S rRNA gene copies in BES (i.e. bulk liquid + biofilm cells) remained almost invariable during operation, except for strains DSM123 and C2T108.3 which experienced a significant decrease, confirming no growth occurred. Collectively, abundance data indicates that monospecific biofilms could be effectively formed and maintained stable for the duration of the experiment.

### Bioelectrochemical production of H_2_ in purple non-sulfur (PNS) bacteria

Inoculation and growth experimental procedure was optimized to enable monospecific biofilm formation on the cathode surface and short-term evaluation of H_2_ production. As stated above, pure electrocatalytic H_2_ production was estimated for every cathode and used as a threshold to determine the effect of the bacterial presence.

**Figure 2:**
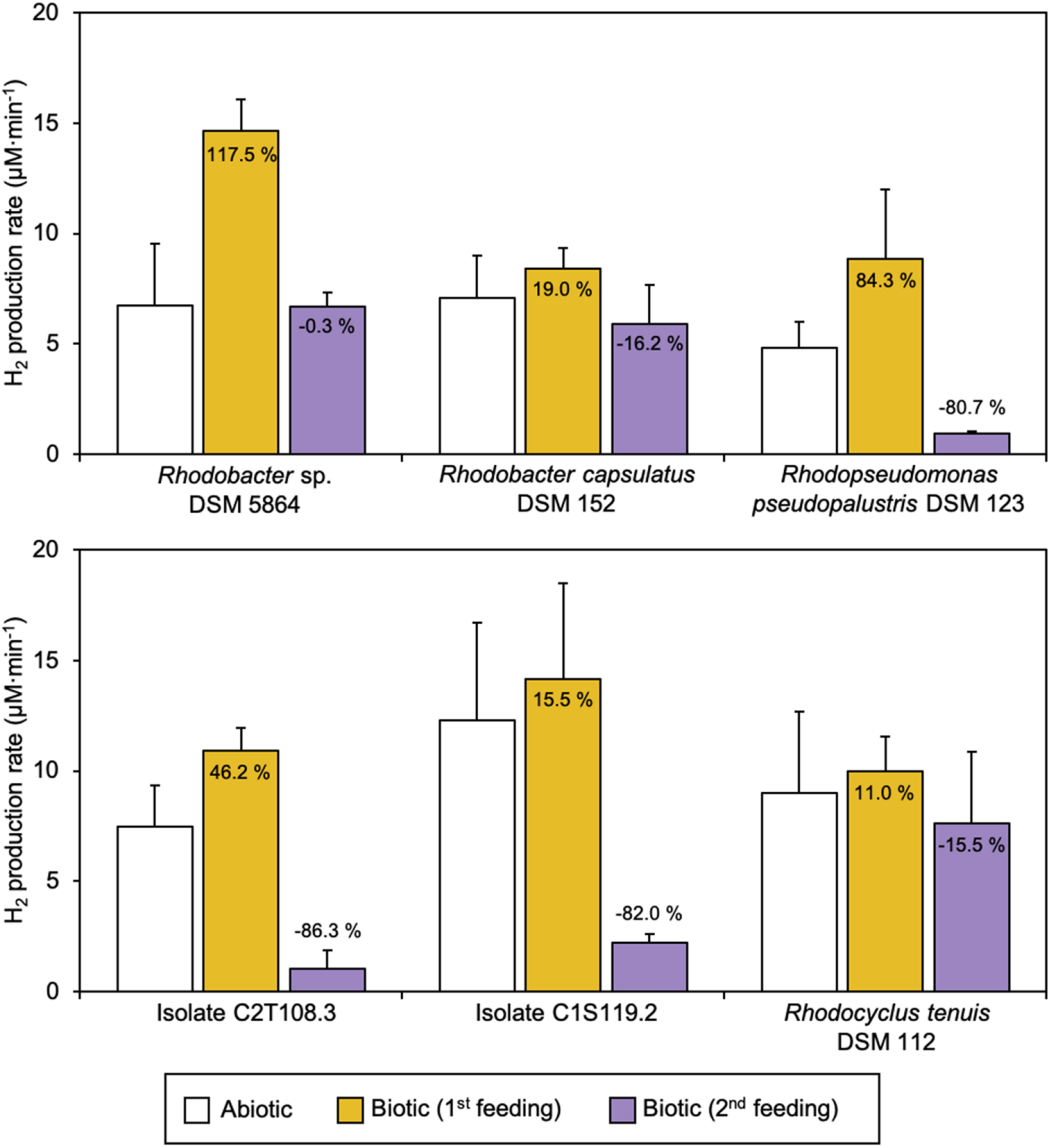
Hydrogen production rates (µM min^−1^) of monospecific biofilms of purple non sulfur (PNS) bacteria after successive CO_2_ feeding in BES reactors. Rates are compared to values obtained for the same electrode in abiotic conditions (white bar) and percentages indicate production rates increase or decrease (negative values).

For all tested PNS strains, H_2_ production started immediately after feeding reactors with CO_2_, and accounted for higher productions (1.2 to 2.2-fold) compared to abiotic conditions (Fig. 2). Specific net productivities (per unit biomass) ranged from 3.6 to 283.3 (µM min^−1^ × 10^7^ copy^−1^ 16S rRNA). In addition, higher current demands and lower energy consumptions were recorded (Table 1). Strain-specific differences were observed after the two CO_2_ feeding performed. In particular, H_2_ production rates for the two *Rhodobacter* sp. (DSM 5864 and DSM 152) decreased to values similar to abiotic conditions (6.7 ± 0.6 and 5.9 ± 1.8 µM min^−1^, respectively). In contrast, for *Rhodopseudomonas* sp. (DSM 123 and isolates C2T108.3 and C1S119.2) and *Rhodocyclus tenuis* biofilms, successive CO_2_ feedings caused a severe decrease of H_2_ production rates (from two to sevenfold compared to maximum production). Differences in current demand and energy consumption occurred accordingly (Table 1). Decreasing H_2_ production rates with *R. pseudopalustris* DSM 123 and isolate C2T108.3 could be explained due to the large decrease (from 1 to 3 magnitude orders) observed in cell densities attached to the cathode over experimental time (Fig. 1). Coulombic efficiencies (CE) for the production of H_2_ remained at similar values to those found in abiotic condition when using the two *Rhodobacter sp*., except after the second CO_2_ feeding for DSM 5864 (CE = 67.8%). Also, when testing *R. tenuis* DSM 112 CE remained similar to abiotic conditions after the second CO_2_ feeding (76.4% and 83.5%, respectively). Contrarily, coulombic efficiencies were severely lower when using *Rhodopseudomonas* sp. in comparison with abiotic conditions (Table 1).

**Table 1:** Maximum H_2_ accumulated, H_2_ production rate, H_2_ net production rate, current demand, energy and coulombic efficiencies (CE %) for the different bacterial strains and experimental conditions.

Bacterial strain	Potential (*vs.* Ag/AgCl)	Experimental conditions	Maximum H_2_ accumulated (µM)	H_2_ production rate (µM min^−1^)	Specific net H_2_ production rate (µM min^−1^ × 10^7^ copy 16S rRNA^−1^)	Current demand (mA m^−2^)	Energy (kWh)	CE (%)
*Rhodobacter* sp. DSM 5864	− 1.0	Abiotic	787.6	6.7 ± 2.8		753.6	2.3 × 10^−6^	95.7
		Biotic (1^st^ feeding)	1897.2	14.6 ± 1.4	14.1 ± 2.5	2397.9	8.0 × 10^−6^	94.8
		Biotic (2^nd^ feeding)	1656.2	6.7 ± 0.6	n.d	1465.6	5.6 × 10^−6^	67.8
*Rhodobacter capsulatus* DSM 152	− 1.0	Abiotic	1355.2	7.1 ± 1.9		962.3	3.3 × 10^−6^	82.8
		Biotic (1^st^ feeding)	2999.0	8.4 ± 0.9	117.2 ± 86.7	817.0	2.4 × 10^−6^	93.4
		Biotic (2^nd^ feeding)	745.9	5.9 ± 1.8	n.d	647.9	1.8 × 10^−6^	83.0
*Rhodopseudomonas pseudopalustris* DSM 123	− 1.0	Abiotic	940.3	4.8 ± 1.2		927.2	2.6 × 10^−6^	85.4
		Biotic (1^st^ feeding)	1173.5	8.9 ± 3.1	66.5 ± 32.1	1675.8	4.5 × 10^−6^	42.7
		Biotic (2^nd^ feeding)	449.8	0.9 ± 0.1	n.d	2400.9	1.6 × 10^−5^	7.9
Isolate C2T108.3	− 1.0	Abiotic	816.8	7.5 ± 1.9		2371.2	1.1 × 10^−5^	80.6
		Biotic (1^st^ feeding)	1773.4	10.9 ± 1.0	7.5 ± 1.9	2994.6	9.3 × 10^−6^	40.8
		Biotic (2^nd^ feeding)	655.8	1.0 ± 0.8	n.d	5603.5	1.8 × 10^−5^	11.1
Isolate C1S119.2	− 1.0	Abiotic	834.6	12.3 ± 4.5		5019.1	4.1 × 10^−5^	83.9
		Biotic (1^st^ feeding)	1726.9	14.2 ± 4.3	283.3 ± 23.2	5097.2	3.1 × 10^−5^	25.5
		Biotic (2^nd^ feeding)	655.8	2.2 ± 0.4	n.d	4610.8	2.6 × 10^−5^	8.3
*Rhodocyclus tenuis* DSM 112	− 1.0	Abiotic	1511.4	9.0 ± 3.7		2436.8	1.3 × 10^−5^	83.5
		Biotic (1^st^ feeding)	4865.5	10.0 ± 1.6	3.6 ± 7.6	2841.6	2.1 × 10^−6^	58.4
		Biotic (2^nd^ feeding)	3459.0	4.5 ± 1.1	n.d	5857.9	3.6 × 10^−5^	76.4
*Sporomusa ovata* DSM 2662	− 1.0	Abiotic	1454.6	4.0 ± 1.3		1505.6	5.8 × 10^−6^	80.7
		Biotic (1^st^ feeding)	123.9	2.1 ± 1.3	n.d	820.8	5.5 × 10^−7^	46.1
		Biotic (2^nd^ feeding)	Not detected		n.d	1228.7	7.8 × 10^−6^	n.d
*Desulfovibrio desulfuricans* DSM 642	− 1.0	Abiotic	938.6	3.5 ± 0.6		1774.6	3.0 × 10^−5^	85.9
		Biotic (1^st^ feeding)	2042.9	13.0 ± 2.9	121.5 ± 25.6	4771.4	1.3 × 10^−5^	73.2
		Biotic (2^nd^ feeding)	1467.3	5.7 ± 1.0	8.0 ± 0.9	4621.9	1.6 × 10^−5^	76.6
	− 0.8	Abiotic	211.5	1.8 ± 0.1		478.0	1.3 × 10^−5^	46.1
		Biotic (1^st^ feeding)	192.7	1.0 ± 0.3	n.d	966.8	2.2 × 10^−6^	28.4
*Desulfovibrio vulgaris* DSM 644	− 1.0	Abiotic	1000.0	3.3 ± 0.9		1758.2	3.0 × 10^−5^	82.8
		Biotic (1^st^ feeding)	1384.7	3.2 ± 0.4	n.d	3245.4	8.4 × 10^−6^	77.0
		Biotic (2^nd^ feeding)	1086.6	4.1 ± 0.6	4.1 ± 1.8	4975.4	3.0 × 10^−5^	17.4
	− 0.8	Abiotic	213.5	2.0 ± 0.8		1038.0	4.0 × 10^−5^	45.8
		Biotic (1^st^ feeding)	124.0	0.8 ± 0.2	n.d	1805.6	2.0 × 10^−5^	15.6
*Desulfovibrio paquesii* DSM 16681	− 1.0	Abiotic	1435.8	5.5 ± 0.6		1107.6	3.7 × 10^−5^	80.8
		Biotic (1^st^ feeding)	3088.7	3.3 ± 2.6	n.d	2525.0	6.6 × 10^−6^	80.5
		Biotic (2^nd^ feeding)	2826.6	45.6 ± 18.8	202.7 ± 91.9	6872.4	3.0 × 10^−5^	91.6
	− 0.8	Abiotic	592.0	1.9 ± 0.1		561.8	1.0 × 10^−6^	50.7
		Biotic (1^st^ feeding)	1232.5	4.5 ± 1.8	13.3 ± 8.3	1241.7	2.6 × 10^−6^	68.4

H_2_ net production rate have been calculated as: (biotic – abiotic rates) per unit 16S rRNA gene. n.d. Not determined.

pH has a great impact on electrochemical performance. According to the Nernst equation and typical solution conditions, an overpotential of − 59 mV is expected per pH unit increase^27^. For that, pH was measured several times during BES operation (Supplementary Table S3). For all the tested bacteria starting pH was between 6.8–7.0. pH plummeted to 5.4–5.7 immediately after sparging pure CO_2_ for over 10 min and increase continuously afterwards. From thermodynamic point of view, such acidic pH reinforced the production of hydrogen. During the experimental period pH followed different trends for each strain. pH decreased to around 5.7–5.8 in the two *Rhodobacter* sp. (DSM 5864 and DSM 152), while for isolate C1S119.1 went up to 7.4 after five days of operation. Similar pHs were measured for the other PNS strains (around 6.6–6.9 at the end of operation). Differences were linked to the rate of bioelectrochemical H_2_ production of each strain and the buffer capacity of each medium (Supplementary Table S4).

Hydrogen production by PNS occurs under photoheterotrophic metabolism^14^. Although several studies have highlighted the metabolic versatility of PNS, focusing mainly in the *in-situ* bioH_2_ production under different substrates, reactor designs and environmental conditions^13,14,28,29^, few studies have been conducted using pure cultures in BES^10,30,31^. Observed changes in H_2_ production rates after different CO_2_ flushes in the reactor when operated with PNS could be due to two opposite effects. First, the possibility that H_2_ was being produced as a residual activity from photo-fermentative growth. This could explain the higher production rates when starting BES operation, but only if some intracellular carbon reservoir had been accumulated during preparation of biofilms. Both *Rhodobacter* and *Rhodopseudomonas* species are able to accumulate polyhydroxy butyrate (PHB) under nutrient starvation (i.e. nitrogen, phosphorus, sulfur) and excess of organic carbon source. Larger amounts of PHB are accumulated under nitrogen limitation and especially when acetate is used as carbon source. These are also the suitable conditions to H_2_ production via photo-fermentation. On the event of a exposure of cells to non-optimal conditions, PHB can be mobilized and used as energy and carbon source^32,33^. Second, an enhanced H_2_ consumption after completed adaptation of cells to the new reactor environment. Although *Rhodobacter* sp. and *Rhodopseudomonas* sp. have been proposed as H_2_-producing bacteria in biocathodes by other authors^10,30^, in the tested conditions these strains did not show such a capacity in the long-term, i.e. H_2_ consumption surpassed production after CO_2_ replenishment. However, current demands increased in three of the tested PNS strains (*R*. *pseudopalustris* DSM 123; isolate C2T108.3 and *R*. *tenuis* DSM 112) after the second CO_2_ feeding (Table 1). This may be indicative of H_2_ being produced and rapidly consumed by the cells. Unfortunately, due to technical limitations for sampling the gas phase, no data for CO_2_ consumption is available to confirm this hypothesis. An alternative would be a direct electron transfer without H_2_ accumulation. In this sense, Bose and co-workers tested *Rhodopseudomonas palustris* strain TIE-1 in cathodes poised at + 0.1 V *vs.* SHE (− 0.1 V *vs.* Ag/AgCl) confirming this strain was able to accept electrons from the poised electrode^34^. Derived electrons from the cathode surface were entering the photosynthetic electron transport chain, leading to a highly reduced cellular environment. Further transcriptomic analysis on the expression levels of *ruBisCO* forms I and II using wild-type strain and a *ruBisCO* double mutant, determined that electron uptake was connected to CO_2_ fixation^31^.

### Bioelectrochemical production of H_2_ in *Sporomusa ovata*

**Figure 3:**
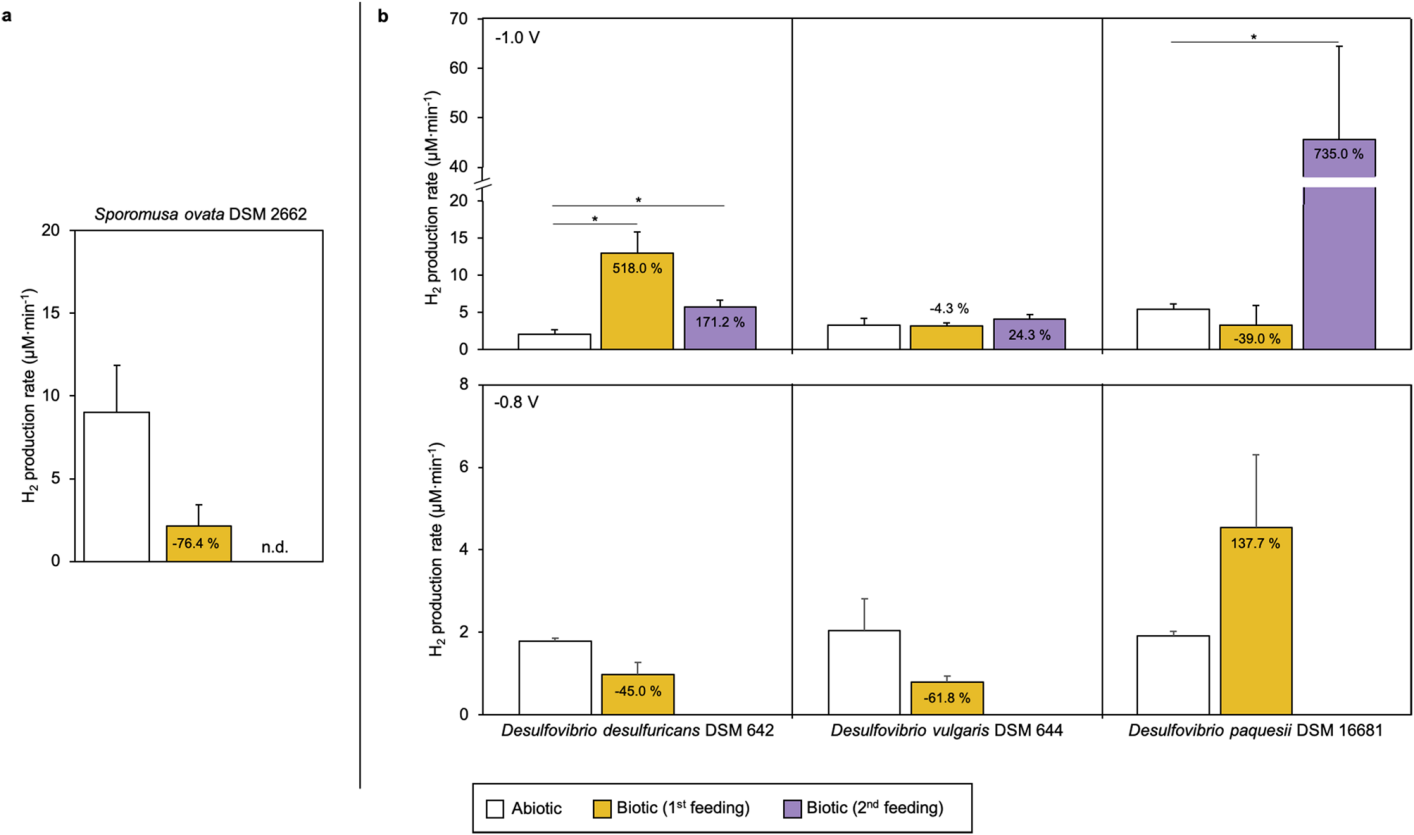
Hydrogen production rates (µM min^−1^) using **a**) *S. ovata* DSM 2662 and **b**) *Desulfovibrio* strains with poised electrodes at − 1.0 V (upper plots) and − 0.8 V *vs.* Ag/AgCl (lower plots). Rates are compared to values obtained for the same electrode in abiotic conditions (white bar) and percentages indicate increase or decrease (negative values) in production rates. Statistically significant differences are shown as * (p < 0.05).

For the system inoculated with *S. ovata* DSM 2662, H_2_ production rate was fourfold lower after the first CO_2_ feeding. Remarkably, after the second feeding no H_2_ production could be detected (Fig. 3a). Coulombic efficiency for the production of H_2_ was lower in biotic than in abiotic conditions (46.1% and 80.7%, respectively). Volatile fatty acids and alcohols were measured during BES operation in all the experimental conditions and tested strains, however only when using *S. ovata* acetate was detected. Acetate concentrations increased over the time reaching 6.4 mM at the end of the operation. Coulombic efficiencies for acetate production increased over operation from 57.2% to 70.3%. Mainly due to acetate production, pH decreased from 7.4 to 6.5 during operation. *Sporomusa* spp. have been widely used in MES^20,35,36^, taking advantage of its homoacetogenic metabolism. Therefore, recorded decreasing H_2_ concentrations in our system could be explained by a quick consumption of H_2_ for acetate biosynthesis. Deutzmann and co-workers found higher H_2_ productions when using cell-free exhausted medium from *Sporomusa sphaeroides* cultures, probably due to the presence of enzymes (such as hydrogenases) into the culture supernatants^20^. More recently, high H_2_ evolving BES at different cathode potentials (from − 0.5 to − 0.9 V *vs.* Ag/AgCl) have been demonstrated by using *S. ovata* cell-free medium due to nickel and cobalt deposition onto the electrode surface^19^. Despite being a frequently studied candidate for bioelectrosynthesis development, and considering our main objective, *Sporomusa* strains seem not to be good candidates for a stable H_2_ producing platform using modified biocathodes. It seems clear that net H_2_ production is only observed in the absence of active microorganisms.

### Bioelectrochemical production of H_2_ in sulphate-reducing bacteria

Sulphate-reducing bacteria can use H_2_ as electron donor while reducing sulphate in anaerobic conditions^37^. But some of them, specially *Desulfovibrio* spp., have been postulated as potentially H_2_ producing microorganisms in BES using pure and mixed cultures^11,16,37^. Here, three different *Desulfovibrio* species were selected as potential candidates to test and compare this process with other phylogenetically distinct bacteria. H_2_ production rates at − 1 V (Fig. 3b) were consistent over time for the three studied species, in agreement with Aulenta and co-workers with *D. paquesii* DSM 16681^16^. H_2_ production rates in *D. desulfuricans* remained significantly higher compared to abiotic conditions (*p* ≤ 0.01) after successive CO_2_ feeding (13 ± 2.9 and 5.7 ± 1.0 µM min^−1^, respectively; Table 1), but a decrease in production rate was observed. While energy consumptions remained similar between feedings (1.3 × 10^−5^ to 1.6 × 10^−5^ kWh), higher current demands were recorded in comparison to abiotic conditions. Coulombic efficiencies for the production of H_2_ remained above 70% being lower than the abiotic (Table 1). At the same potential, *D. vulgaris* did not increase production rates when compared to abiotic conditions (3.3 ± 0.9 and 3.2 ± 0.4 – 4.1 ± 0.6 µM min^−1^, respectively) although a higher current demand was measured (Table 1). The highest increase in H_2_ production was observed for *D. paquesii* after two CO_2_ flushes in the system operated at − 1 V. A significant eightfold increase (*p* = 0.02) in H_2_ production rate compared to abiotic conditions (5.5 ± 0.6 and 45.6 ± 18.8 µM min^−1^, respectively) was observed, leading to an increased current demand but a slightly lower energy consumption. Similarly as exposed by Aulenta and co-workers, coulombic efficiencies (Table 1) remained between 80–100%^16^. For all the tested *Desulfovibrio* starting pH was 7.7 and during BES operation pH decreased to 6.2–7.2 (Supplementary Table S3).

Since two out of the three *Desulfovibrio* strains showed the highest capacity in net H_2_ production among the ten tested strains, experiments at less reducing potentials (− 0.8 and − 0.6 V *vs.* Ag/AgCl; Fig. 3) were also performed. When biocathodes were poised at − 0.8 V, measured current demands increased from abiotic to biotic conditions with all *Desulfovibrio* strains (Table 1). Similar H_2_ production rates were found for *D. desulfuricans* and *D. vulgaris*, being slightly lower than in abiotic conditions. Otherwise, in the presence of *D. paquesii* DSM 16681, higher net H_2_ production rate was obtained. H_2_ production was not detected neither in abiotic nor biotic conditions at − 0.6 V *vs.* Ag/AgCl. Biocathodes in the presence of *D. paquesii* DSM 16681 had been characterized before at − 0.9 and − 0.7 V *vs.* Ag/AgCl by Aulenta and co-workers^16^. The authors hypothesized that *Desulfovibrio* could not use efficiently the electrons when cathodes were polarized at potentials above − 0.9 V because the recorded current demands in these conditions were very similar at the ones obtained in abiotic conditions. In contrast, our results suggested that, at least at − 0.8 V *vs.* Ag/AgCl, *D. paquesii* was able to produce H_2_ at higher rates and, with regard to the higher current demand, usage of electrons from the cathode was confirmed.

The obtained results demonstrated that at least *D. desulfuricans* and *D. paquesii* were able to increase H_2_ production in biocathodes at the used experimental conditions. *D. paquesii* was reported before as an electroactive microorganism able to produce H_2_ highly efficiently with production rates from five to tenfold times higher than in abiotic conditions, and coulombic efficiencies near 100%^16^. Also, *D. caledoniensis* has been proved as a H_2_ producing catalyst at − 0.8 V *vs.* Ag/AgCl^17^. Conversely, in the tested conditions no conclusive results could be obtained for *D. vulgaris*. However, direct evidence on electrocatalytic H_2_ production when using purified *D. vulgaris* [Fe] hydrogenase was obtained when coating cathode electrodes with this enzyme^38^. Also, a stable hydrogen production using whole cells of *D. vulgaris* have been reported^37^, confirming electroactivity of the microorganism in the presence of methyl viologen and electrodes poised at − 0.7 V *vs.* Ag/AgCl. Although not tested here, the presence of soluble electron shuttles, such as methyl viologen, may impact the H_2_ production rate, but of course the use of these compounds will impose additional parameters to be controlled in order to develop stable H_2_ production platforms for BES.

### Electrochemical characterization

**Figure 4:**
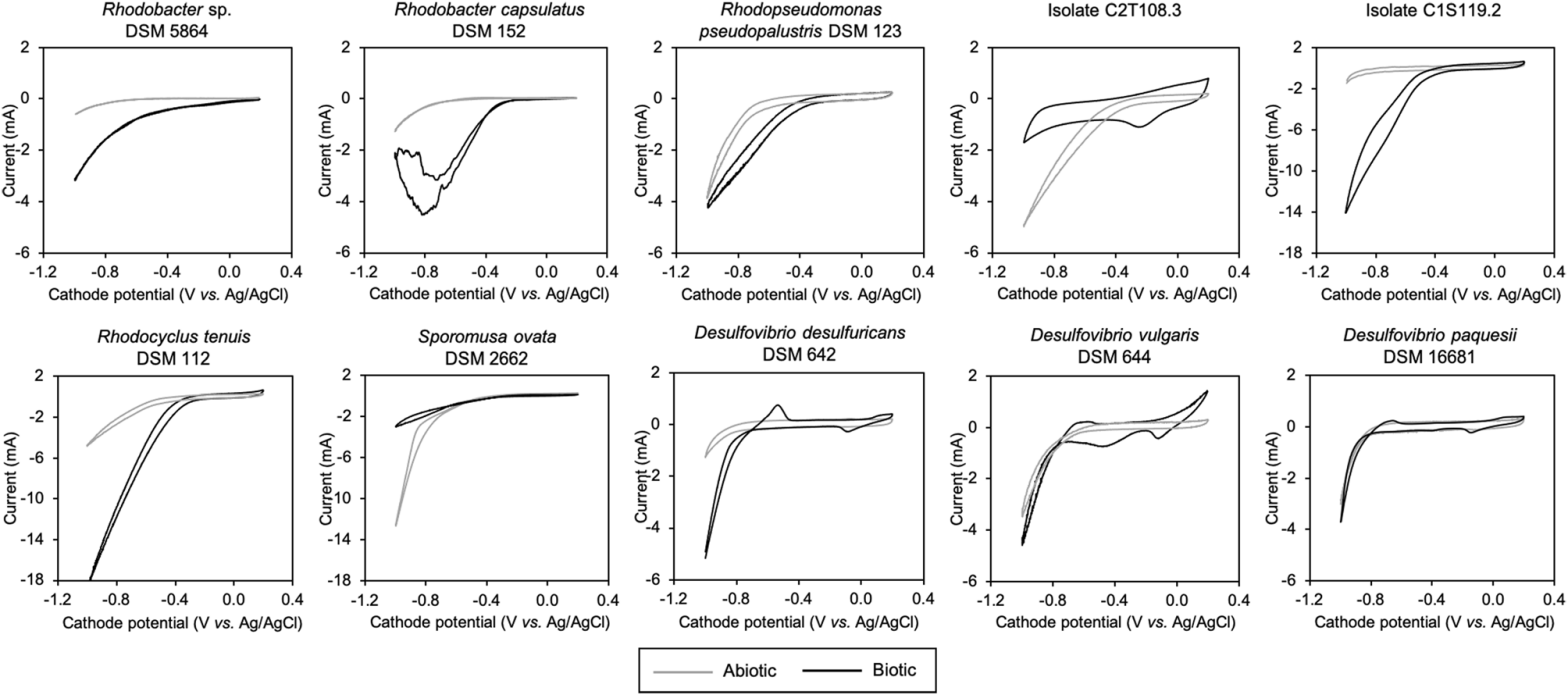
Cyclic voltammetries (CV) for *Rhodobacter*, *Rhodopseudomonas*, *Rhodocyclus*, *Sporomusa* and *Desulfovibrio* strains. Representative voltammograms in abiotic cathode electrodes (grey) and biocathodes (black) are shown.

Biofilms were characterized electrochemically using cyclic voltammetries (CV) after 5 days of operation and compared to abiotic CVs (Fig. 4). In eight out of the ten tested strains, the current demand of biotic CVs differed significantly from the abiotic control indicating that those biofilms were electroactive. Current demand started to increase around − 0.7 to − 0.8 V *vs.* Ag/AgCl in abiotic conditions while in the presence of DSM 5864, DSM 152, DSM 123, C1S119.2, and DSM 112 increased current demands were recorded at higher potentials, from − 0.4 to − 0.6 V *vs.* Ag/AgCl. This was interpreted as an indication that the biofilm catalyzed the reduction reaction of protons (H^+^) decreasing energy losses that could be associated to the catalytic hydrogen production. Highest current demands were observed for C1S119.2 and DSM 112 reaching 14.0 and 18.3 mA, respectively. Even though, these results could not be linked to a higher net H_2_ production rates. It should be considered that CV measurements are highly sensitive and can detect small induced changes in the redox state of the cell that may not be sustained in the long run.

A redox pair was identified at − 0.65 V *vs.* Ag/AgCl for *Rhodobacter* sp. DSM 5864, *R. pseudopalustris* DSM 123, isolate C1S119.2 and *Desulfovibrio* spp., which has been typically observed in H_2_-producing biocathodes^9,10^ and related to the *D. vulgaris* [Fe] hydrogenase activity^38^. Similar results as the presented with *Desulfovibrio* species were found by other authors using pure cultures of *D. caledoniensis* and *D. paquesii*^16,17^. Redox peaks could not be clearly identified for the experiments with *R. capsulatus* DSM 152, *R. tenuis* DSM 112 and *S. ovata*. An additional reduction peak was found at − 0.46 V when using *D. vulgaris*. also found in electrochemical experiments conducted with *D. gigas* [NiFe] hydrogenases in bulk suspension^39^. Although electrochemical characterization using CV revealed some electroactivity for almost all the strains, unfortunately these could not be translated into direct H_2_ production, except for *D. desulfuricans* DSM 642 and *D. paquesii* DSM 16681. Defined co-cultures where hydrogenotrophic bacteria could be sustained by an efficient biological hydrogen producer has been highlighted before as an important step forward to improve microbial electrosynthesis^40^. According to our results, *Desulfovibrio* species are the best candidates, among all tested strains, to further develop the use of biofilm coated cathodes as a stable H_2_ production platform in microbial electrosynthesis.

## Conclusions

In this study we presented an evaluation of ten different bacteria (including species of *Rhodobacter*, *Rhodopseudomonas*, *Rhodocyclus*, *Sporomusa* and *Desulfovibrio*) as a first step in the development of a stable H_2_-evolving platform for microbial electrosynthesis. All used strains and isolates had been previously proved as effective H_2_ producers in bioelectrochemical systems by different authors. In this work, we tested all strains using an optimized and identical protocol based on the development of monospecific biofilms, thus facilitating comparisons among them. Cell densities measured by qPCR revealed that cells had the tendency to attach to the electrode surface in BES reactors, independently on their ability to produce H_2_. Hydrogen production rates increased compared to abiotic conditions in all tested strains except *S. ovata* DSM 2662. In four of them, *R. capsulatus* DSM 152, isolate C1S119.2, *D. desulfuricans* DSM 642, *D. paquesii* DSM 16681, specific H_2_ production rates were markedly higher but only on *Desulfovibrio* strains were sustained in the long term. This fact, together with a major stability of the biofilms of these species, resulted in *D. desulfuricans* DSM 642 and *D. paquesii* DSM 16681 as the most promising candidates to evolve selective biologic H_2_-producing cathodes. Despite differences in production rates, eight strains presented some electroactivity according to cyclic voltammetry measurements and are candidates to additional explorations of their performance in BES under changing conditions. Our results represent a significant step forward to further study H_2_-producing bacteria into defined co-cultures for microbial electrosynthesis and electro-fermentation.

## Materials and methods

### Bacterial strains and maintenance conditions

All bacterial strains used in this study were obtained from Leibniz Institute DSMZ – German Collection of Microorganisms and Cell Cultures, except for isolates C2T108.3 and C1S119.2 obtained from a denitrifying biocathode and tentatively identified as *Rhodopseudomonas*^30^. *Rhodobacter* sp. DSM 5864, *Rhodobacter capsulatus* DSM 152, *Rhodopseudomonas pseudopalustris* DSM 123, *Rhodocyclus tenuis* DSM 112 and isolates C2T108.3 and C1S119.2 were cultured using DSM 27 medium. *Sporomusa ovata* DSM 2662 was routinely cultured on DSM 311 medium and *Desulfovibrio paquesii* DSM 16681, *Desulfovibrio vulgaris* DSM 644, *Desulfovibrio desulfuricans* DSM 642 using DSM 63 medium. All cultures were grown at recommended culturing conditions following the instructions provided by the DSMZ. Culture inoculation and maintenance was done into serum bottles with butyl rubber septa under anaerobic conditions. All manipulations were carried out in an anaerobic chamber (gas mixture N_2_:H_2_:CO_2_ [90:5:5], COY Laboratory Products, INC, USA).

### Biofilm development on carbon cloth electrodes

In order to stimulate formation of monospecific biofilms on carbon cloth, slight modifications of culture conditions were applied (Supplementary Tables S5–S7). Inclusion of one source of organic matter was done as an adaptation for autotrophic conditions finally used during BES operation. Modifications of DSM 27 included: substitution of NH_4_-acetate for Na-acetate (0.53 g L^−1^) and exclusion of yeast extract, Na-succinate, Na-resazurin solution and NH_4_Cl. pH was set to 6.8, and medium was degasified with helium (He) to reduce the presence of N_2_ into the medium. All modifications were carried out to avoid product inhibition of the nitrogenase activity, and force bacteria to get rid of the excess energy and reducing power through H_2_ production. Modifications of DSM 311 medium included: exclusion of casitone, betaine, L-cysteine-HCl, Na_2_S and Na-resazurin, solution was set at pH 7 and degasified with nitrogen (N_2_). Modified medium specially designed was used for *Desulfovibrio* species^16^. For all strains and isolates, brand-new treated carbon cloth electrodes were immersed in freshly prepared media modified to enhance bacterial growth and biofilm formation. Incubations were performed in 100 mL serum bottles. *Rhodobacter*, *Rhodopseudomonas* (including isolates C2T108.3 and C1S119.2) and *Rhodocyclus* species were incubated under constant light at 25 °C, while *Sporomusa ovata* and *Desulfovibrio* sp. were maintained in the dark and 30 °C. As for bacterial strains maintenance, all manipulations were carried out under anaerobic conditions. Biofilms were allowed to grow on the surface of electrodes for a minimum of 10 days.

### Bioelectrochemical system setup and operation

H-type bioelectrochemical systems (BES) reactors with a nominal capacity of 150 mL (Adams & Chittenden Scientific Glass, Berkeley CA – USA) were used. Each one consisted of two chambers, anodic and cathodic, separated by a cation exchange membrane (21.2 cm^2^ surface area; CMI-7000, Membranes International Inc, USA). Carbon cloth (NuVant’s ELAT LT2400W, FuelCellsEtc USA) with 24 cm^2^ surface area connected directly to a stainless-steel wire (AISI 304 Grade and 1 mm thickness) was used as cathode electrode (working electrode). The contribution of the wire to H_2_ production was considered to be negligible. An Ag/AgCl reference electrode (+ 197 mV *vs.* SHE, sat KCl, SE11 Sensortechnik Meinsberg, Germany) was placed into the cathodic chamber. A graphite rod (5 × 250 mm, MERSEN IBERICA, Spain) was used as anode (counter electrode) (Supplementary Fig. S1). Prior to usage, carbon cloth pieces meant to be used as electrodes were cleaned with 0.5 M HCl, 0.5 M NaOH and miliQ water for 12 h each solution to remove impurities. Chronoamperometric experiments were performed in abiotic and biotic conditions at − 1.0, − 0.8 and − 0.6 V *vs.* Ag/AgCl using a potentiostat (BioLogic, Model VSP, France). All the potentials indicated in this work are relative to Ag/AgCl. H-type cells were maintained at 30 ± 2 °C, with constant stirring by means of a magnetic bar at 200 rpm (MultiMix D9 P V1, OVAN, Spain) and in the dark.

Anodic and cathodic chambers were filled with the corresponding modified inorganic media (Supplementary Tables S5–S7). Abiotic (cell-free) cathodes poised at − 1.0 V *vs.* Ag/AgCl were used to test BES set-up. After 5–6 h of operation, H_2_ saturation was reached (~ 800 µM, in view of the media composition and reactor temperature). Slight increases from saturation value were recorded over time if overpressure was allowed to the system. The presence of leaks in the reactor were tested after disconnecting the potentiostat and recording the H_2_ concentration decrease. Measured leaks did not exceed 3.5 µM h^−1^.

After abiotic tests were performed, the same electrode was incubated in the presence of bacteria until a biofilm was formed (see previous subsection). Carbon cloth electrodes with a monospecific biofilm formed on its surface were placed directly into the cathodic chamber. Remaining cells into the supernatants (90 mL) were harvested in the late exponential phase at an optical density (OD_600_) of 0.3–0.4, pelleted by centrifugation (4,400 rpm, 15 min, 4 °C), resuspended into 1 mL of inorganic modified medium and added into the cathodic chamber. Headspace was saturated with filter-sterilized pure CO_2_ at the beginning of the operation. Biotic experiments lasted 5 days. Once set-up was completed, H_2_ production rates were re-evaluated and compared to abiotic tests using the same operational conditions (cathodic voltage − 1.0 V *vs.* Ag/AgCl) and maintained for 3 days. After this, reactor headspace was flushed with a filter-sterilized pure CO_2_ stream, and production re-evaluated for two additional days (second CO_2_ feeding). Flushing was repeated at day 5 to ensure inorganic carbon source availability before cyclic voltammetries were done.

### Electrochemical characterization and calculations

*On-line* hydrogen concentration measurements were performed using a H_2_ NP-500 microsensor (Unisense, Denmark) directly placed in the liquid compartment close to the cathode surface. Microsensors were regularly calibrated using a saturated water solution using CO_2_:H_2_ gas mixture (80:20% v/v) following the specifications of the manufacturer. Liquid samples from the cathodic chamber were taken during biotic operation to control pH and volatile fatty acids (i.e. acetate), and alcohols (i.e. ethanol) concentration. VFA and alcohols were analyzed using a gas chromatograph Agilent 7890A (Agilent Technologies, US) equipped with a DB-FFAP column and a flame ionization detector. pH was measured with a pH meter (pH meter Basic 20, Crison Instruments, Spain). After liquid sample extraction, withdrawn volumes were replaced with freshly prepared medium.

Cyclic voltammetries (CV) were performed to confirm electrochemical activity. The technique allowed the characterization of electroactive biofilms analyzing changes in the slope of current *vs.* cathode potential curves, and estimate cathode potentials at which redox reactions are taking place^41^. CVs were performed using EC-Lab v10.37 software (Bio-Logic Science Instruments, France). Four cycles were done within a range of 0.2 V to − 1.0 V and at a scan rate of 1.0 mV s^−1^. The obtained CV signals in biotic conditions were compared to abiotic ones. Raw CV data were used for oxidative-reductive peak detection by calculating the first derivative. Analyses were performed using the free-software QSoas^42^. The mid-point potential (Ef) of redox couples was calculated as the mean value of the oxidative and reductive potential.

Ionic losses were calculated for each medium used. The ionic loss (mV) is related to the electrolyte resistance of the anolyte and catholyte and was estimated according to Ter Heijne et al.^43^.

During chronoamperometrical operation, power (*P*, Eq. 1) and energy requirements (*E*, Eq. 2) were calculated as shown in Eqs. (1) and (2),

1 $$P = I\cdot V$$

2 $$E = P\cdot t$$

being *I* intensity, and *V* voltage.

Columbic efficiency (CE) was calculated according to Patil et al.^44^ (Eq. 3). *Ci* is the compound *i* concentration in the liquid phase (mol *Ci* L^−1^), *n*_*i*_ is the molar conversion factor (2 and 8 eq. mol^−1^ for H_2_ and acetate, respectively), *F* is Faraday’s constant (96,485 C mol e^−1^), *V* (L) is the net liquid volume of the cathode compartment, and *I* is the intensity demand of the system (A).

3 $${\text{CE}}\left( \% \right) = \frac{{C_{i} \cdot\mathop \sum \nolimits_{i} n_{i} \cdot F\cdot V }}{{\mathop \smallint \nolimits_{0}^{t} I\cdot dt}} \times 100$$

H_2_ production rates in all conditions and strains were calculated as a linear response covering the first 25 min of operation according to Tremblay and co-workers^19^. Linear regressions were calculated for this time-period using SigmaPlot version 11.0 (Systat Software, USA, www.systatsoftware.com) and H_2_ production rates were obtained from the slope. Unpaired t-tests were used to evaluate statistical significance between biotic and abiotic H_2_ production rates, current demand, or energy consumption.

### DNA extractions and 16S rRNA gene determinations

Samples from both biofilm and bulk liquid were collected under anaerobic conditions during the growth of bacteria and under BES operation. For biofilm measurements, pieces of carbon cloth electrode (1.5 cm^2^ each) were taken directly using sterile forceps and scissors. For bulk measurements, 10 mL samples were centrifuged (4,400 rpm, 15 min, 4 °C) and supernatants discarded. Both electrode and pelleted cells were stored at − 20 °C until DNA extraction.

DNA extraction was performed using a cetyltrimethylammonium bromide (cTAB) based protocol^45^. DNA concentrations were measured using Qubit 2.0 Fluoremeter (Thermo Fisher Scientific, USA). Previous to qPCR amplification, samples with 1 ng µL^−1^ or higher were diluted to avoid inhibition due to excess of DNA. qPCR was used to quantify DNA gene copies targeting 16S rRNA in each sample using 341F and 534R primer pair following the conditions described by López-Gutiérrez and co-workers^46^. Reactions were performed using the LightCycler 480 SYBR Green I Master Mix (Roche Life Science, Switzerland) and a Lightcycler 96 Real-Time PCR instrument. In all cases, two sample volumes, 1 and 2 µL in a 20 µL total volume were used to ensure no inhibition occurred. A tenfold dilutions series (10^3^–10^7^ copies/mL) of a linearized plasmid containing a 16S rRNA gene sequence was used as standard curve. In all cases qPCR efficiencies were above 90%.

Gene copies per unit mL or cm^2^ in bulk and biofilm samples were calculated considering dilutions and initial sample volume or surface.

## Supplementary information

Supplementary Information 1.

## References

[1] Nevin KP, Woodard TL, Franks AE, Summers ZM, Lovley DR (2010). Microbial electrosynthesis: Feeding microbes electricity to convert carbon dioxide and water to multicarbon extracellular organic compounds. MBio.

[2] Lovley DR, Nevin KP (2013). Electrobiocommodities: powering microbial production of fuels and commodity chemicals from carbon dioxide with electricity. Curr. Opin. Biotechnol..

[3] Choi O, Sang B-IBI (2016). Extracellular electron transfer from cathode to microbes: application for biofuel production. Biotechnol. Biofuels.

[4] Lovley DR (2012). Electromicrobiology. Annu. Rev. Microbiol..

[5] Pous N (2015). Monitoring and engineering reactor microbiomes of denitrifying bioelectrochemical systems. RSC Adv..

[6] Perona-Vico E, Blasco-Gómez R, Colprim JS, Puig S, Bañeras L (2019). [NiFe]-hydrogenases are constitutively expressed in an enriched *Methanobacterium* sp. population during electromethanogenesis. PLoS ONE.

[7] Logan BE, Rossi R, Ragab A, Saikaly PE (2019). Electroactive microorganisms in bioelectrochemical systems. Nat. Rev. Microbiol..

[8] Kracke F, Vassilev I, Krömer JO (2015). Microbial electron transport and energy conservation—the foundation for optimizing bioelectrochemical systems. Front. Microbiol..

[9] Batlle-Vilanova P (2014). Assessment of biotic and abiotic graphite cathodes for hydrogen production in microbial electrolysis cells. Int. J. Hydrogen Energy.

[10] Puig S (2017). Tracking bio-hydrogen-mediated production of commodity chemicals from carbon dioxide and renewable electricity. Bioresour. Technol..

[11] Jourdin L, Freguia S, Donose BC, Keller J (2015). Autotrophic hydrogen-producing biofilm growth sustained by a cathode as the sole electron and energy source. Bioelectrochemistry.

[12] Liu Z, Wang K, Chen Y, Tan T, Nielsen J (2020). Third-generation biorefineries as the means to produce fuels and chemicals from CO2. Nature Catalysis.

[13] Ergal İ (2018). The physiology and biotechnology of dark fermentative biohydrogen production. Biotechnol. Adv..

[14] Koku H, Erolu I, Gunduz U, Yucel M, Turker L (2002). Aspect of the metabolism of hydrogen production by *Rhodobacter sphaeroides*. Int J Hydrog. Energy.

[15] Geelhoed JS, Stams AJM (2011). Electricity-assisted biological hydrogen production from acetate by *Geobacter sulfurreducens*. Environ. Sci. Technol..

[16] Aulenta F, Catapano L, Snip L, Villano M, Majone M (2012). Linking bacterial metabolism to graphite cathodes: electrochemical insights into the H2-producing capability of *Desulfovibrio* sp. Chemsuschem.

[17] Yu L, Duan J, Zhao W, Huang Y, Hou B (2011). Characteristics of hydrogen evolution and oxidation catalyzed by *Desulfovibrio caledoniensis* biofilm on pyrolytic graphite electrode. Electrochim. Acta.

[18] Croese E, Pereira MA, Euverink GJ, Stams AJ, Geelhoed JS (2011). Analysis of the microbial community of the biocathode of a hydrogen-producing microbial electrolysis cell. Appl. Microbiol. Biotechnol..

[19] Tremblay PL, Faraghiparapari N, Zhang T (2019). Accelerated h2 evolution during microbial electrosynthesis with *Sporomusa ovata*. Catalysts.

[20] Deutzmann JS, Sahin M, Spormann AM (2015). Extracellular enzymes facilitate electron uptake in biocorrosion and bioelectrosynthesis. MBio.

[21] Lienemann M, Deutzmann JS, Milton RD, Sahin M, Spormann AM (2018). Mediator-free enzymatic electrosynthesis of formate by the *Methanococcus maripaludis* heterodisulfide reductase supercomplex. Bioresour. Technol..

[22] Kundu A, Sahu JN, Redzwan G, Hashim MA (2013). An overview of cathode material and catalysts suitable for generating hydrogen in microbial electrolysis cell. Int. J. Hydrogen Energy.

[23] Kracke F (2019). Robust and biocompatible catalysts for efficient hydrogen-driven microbial electrosynthesis. Commun. Chem..

[24] Call D, Logan BE (2008). Hydrogen production in a single chamber microbial electrolysis cell lacking a membrane. Environ. Sci. Technol..

[25] Selembo PA, Merrill MD, Logan BE (2010). Hydrogen production with nickel powder cathode catalysts in microbial electrolysis cells. Int. J. Hydrogen Energy.

[26] Acinas SG, Marcelino LA, Klepac-Ceraj V, Polz MF (2004). Divergence and redundancy of 16S rRNA sequences in genomes with multiple rrn operons. J. Bacteriol..

[27] Clauwaert P (2008). Minimizing losses in bio-electrochemical systems: The road to applications. Appl. Microbiol. Biotechnol..

[28] Carlozzi P, Lambardi M (2009). Fed-batch operation for bio-H2 production by Rhodopseudomonas palustris (strain 42OL). Renew. Energy.

[29] Gebicki J, Modigell M, Schumacher M, Van Der Burg J, Roebroeck E (2010). Comparison of two reactor concepts for anoxygenic H2 production by *Rhodobacter capsulatus*. J. Clean. Prod..

[30] Vilar-Sanz A (2018). Denitrifying nirK-containing alphaproteobacteria exhibit different electrode driven nitrite reduction capacities. Bioelectrochemistry.

[31] Guzman MS (2019). Phototrophic extracellular electron uptake is linked to carbon dioxide fixation in the bacterium *Rhodopseudomonas palustris*. Nat. Commun..

[32] Kim MS, Kim DH, Cha J, Lee JK (2012). Effect of carbon and nitrogen sources on photo-fermentative H2 production associated with nitrogenase, uptake hydrogenase activity, and PHB accumulation in *Rhodobacter sphaeroides* KD131. Bioresour. Technol..

[33] Wu SC, Liou SZ, Lee CM (2012). Correlation between bio-hydrogen production and polyhydroxybutyrate (PHB) synthesis by *Rhodopseudomonas palustris* WP3-5. Bioresour. Technol..

[34] Bose A, Gardel EJ, Vidoudez C, Parra EA, Girguis PR (2014). Electron uptake by iron-oxidizing phototrophic bacteria. Nat. Commun..

[35] Aryal N, Tremblay PL, Lizak DM, Zhang T (2017). Performance of different Sporomusa species for the microbial electrosynthesis of acetate from carbon dioxide. Bioresour. Technol..

[36] Nevin KP (2011). Electrosynthesis of organic compounds from carbon dioxide is catalyzed by a diversity of acetogenic microorganisms. Appl. Environ. Microbiol..

[37] Lojou E, Durand MC, Dolla A, Bianco P (2002). Hydrogenase activity control at *Desulfovibrio vulgaris* cell-coated carbon electrodes: biochemical and chemical factors influencing the mediated bioelectrocatalysis. Electroanalysis.

[38] Guiral-Brugna M, Giudici-Orticoni M-T, Bruschi M, Bianco P (2001). Electrocatalysis of the hydrogen production by [Fe] hydrogenase from *Desulfovibrio vulgaris* Hildenborough. J. Electroanal. Chem..

[39] Cordas CM, Moura I, Moura JJG (2008). Direct electrochemical study of the multiple redox centers of hydrogenase from *Desulfovibrio gigas*. Bioelectrochemistry.

[40] Deutzmann JS, Spormann AM (2017). Enhanced microbial electrosynthesis by using defined co-cultures. ISME J..

[41] Harnisch F, Freguia S (2012). A basic tutorial on cyclic voltammetry for the investigation of electroactive microbial biofilms. Chem. Asian J..

[42] Fourmond V (2016). QSoas: a versatile software for data analysis. Anal. Chem..

[43] Ter Heijne A, Hamelers HVM, De Wilde V, Rozendal RA, Buisman CJN (2006). A bipolar membrane combined with ferric iron reduction as an efficient cathode system in microbial fuel cells. Environ. Sci. Technol..

[44] Patil SA (2015). A logical data representation framework for electricity-driven bioproduction processes. Biotechnol. Adv..

[45] Llirós M, Casamayor EO, Borrego C (2008). High archaeal richness in the water column of a freshwater sulfurous karstic lake along an interannual study. FEMS Microbiol. Ecol..

[46] López-Gutiérrez JC (2004). Quantification of a novel group of nitrate-reducing bacteria in the environment by real-time PCR. J. Microbiol. Methods.

