# Supplementary material for: Bacteria coated cathodes as an in-situ hydrogen evolving platform for microbial electrosynthesis
Source: Sci Rep. 2020 Nov 16;10:19852. doi: 10.1038/s41598-020-76694-y (PMC7670457; doi:10.1038/s41598-020-76694-y)
Supplement: Supplementary file 1 — Supplementary Information 1. [file 41598_2020_76694_MOESM1_ESM.pdf]

Bacteria coated cathodes as an *in-situ* hydrogen evolving platform for microbial electrosynthesis

Elisabet Perona-Vico[1], Laura Feliu-Paradeda[1], Sebastià Puig[2], Lluis Bañeras[1*]

[1] Dr. L. Bañeras, L. Feliu-Paradeda, E. Perona-Vico
Molecular Microbial Ecology Group, Institute of Aquatic Ecology
University of Girona
Maria Aurèlia Capmany 40, 17003 Girona (Spain)
E-mail: lluis.banyeras@udg.edu

[2] Dr. S. Puig
LEQUiA, Institute of the Environment
University of Girona
Maria Aurèlia Capmany 69, 17003 Girona (Spain)

* Correspondence to lluis.banyeras@udg.edu

Additional Results

Supplementary Table S1. Hydrogen production rates, current demand and energy consumption in abiotic conditions at different cathode potentials with the three used inorganic media modified for BES operation.

Medium	Cathode potential (V *vs.* Ag/AgCl)	H_2 production rate ($\mu M \cdot min^{-1}$)	Current demand ($mA \cdot m^{-2}$)	Energy (kWh)
Modified DSM 27	-0.6	n.d.	-	-
	-0.8	2.3 ± 0.8	689.6 ± 387.1	$6.0 \cdot 10^{-6} \pm 4.5 \cdot 10^{-6}$
	-1.0	8.4 ± 3.0	1749.2 ± 894.4	$1.2 \cdot 10^{-5} \pm 6.0 \cdot 10^{-6}$
Modified DSM 311	-0.6	n.d.	-	-
	-0.8	2.1 ± 0.7	644.9 ± 75.6	$5.1 \cdot 10^{-6} \pm 2.4 \cdot 10^{-6}$
	-1.0	6.4 ± 1.8	1626.2 ± 125.9	$3.8 \cdot 10^{-5} \pm 1.0 \cdot 10^{-5}$
Modified medium based on Aulenta *et al.* [1]	-0.6	n.d.	-	-
	-0.8	1.9 ± 0.6	781.0 ± 213.2	$4.6 \cdot 10^{-5} \pm 3.3 \cdot 10^{-6}$
	-1.0	5.1 ± 0.8	1534.7 ± 105.2	$2.7 \cdot 10^{-5} \pm 1.3 \cdot 10^{-5}$

n.d. Hydrogen was not detected.

Supplementary Table S2. Ionic losses (mV) for each of the three used media modified for BES operation.

Media	I_{ions} ($A \cdot m^{-2}$)	d_{an} (m)	d_{cat} (m)	A_{an} (m^2)	A_{cat} (m^2)	$Cond_{an}$ ($S \cdot cm^{-1}$)	$Cond_{cat}$ ($S \cdot cm^{-1}$)	Ionic loss (mV)
Modified DSM 27	1.078	0.0375	0.045	0.0014	0.0024	0.22	0.22	+184
Modified DSM 311	1.545	0.0375	0.045	0.0014	0.0024	0.68	0.68	+85
Modified medium based on Aulenta *et al.* [1]	1.050	0.0375	0.045	0.0014	0.0024	0.44	0.44	+89

I_{ions}: Current density.
d_{an}, d_{cat}: Distance between the anode/cathode and the membrane.
A_{an}, A_{cat}: Surface area.
$Cond_{an}$, $Cond_{cat}$: Conductivity.

Supplementary Table S3. pH during BES operation. Measurements were performed before CO2 feeding procedure.

Bacterial strain	Day of operation		
	**0**	**3**	**5**
Rhodobacter sp. DSM 5864	6.79	5.79	5.65
Rhodobacter capsulatus DSM 152	6.80	6.08	5.75
Rhodopseudomonas pseudopalustris DSM 123	6.91	6.33	6.55
Isolate C2T108.3	6.86	7.30	6.73
Isolate C1S119.2	6.80	7.48	7.41
Rhodocyclus tenuis DSM 112	6.99	7.11	6.91
Sporomusa ovata DSM 2662	7.41	6.54	6.51
Desulfovibrio desulfurican DSM 642	7.66	7.68	7.18
Desulfovibrio vulgaris DSM 644	7.67	6.92	6.77
Desulfovibrio paquesii DSM 16681	7.70	7.38	6.17

Supplementary Table S4. pH values recorded after ten minutes of CO_2 bubbling for each medium used for BES operation.

	**Inorganic DSM 27**	**Inorganic DSM 311**	**Inorganic Aulenta *et al.*, 2012**
**Initial**	6.8	7.0	7.0
**After CO_2 bubbling**	5.7	5.4	5.7

Additional Materials and Methods

Supplementary Table S5. Modification to medium DSM 27 during biofilm formation and BES operation with *Rhodobacter* sp., *Rhodopseudomonas* sp. and *Rhodocyclus* sp.

	Biofilm formation	BES operation
Component	Amount	Amount
(Na)-acetate	0.53 g·L^{-1}	----
KH_2PO_4	0.50 g·L^{-1}	0.50 g·L^{-1}
$MgSO_4 \cdot 7H_2O$	0.40 g·L^{-1}	0.40 g·L^{-1}
NaCl	0.40 g·L^{-1}	0.40 g·L^{-1}
$CaCl_2 \cdot 2H_2O$	0.05 g·L^{-1}	0.05 g·L^{-1}
Vitamin B_{12} solution	0.40 mL·L^{-1}	0.40 mL·L^{-1}
Trace element solution SL-6	1.00 mL·L^{-1}	1.00 mL·L^{-1}
L-Cysteine-HCl · H_2O	0.30 g·L^{-1}	0.30 g·L^{-1}

Supplementary Table S6. Modification to medium DSM 311 during biofilm formation and BES operation with *Sporomusa ovata*.

	Biofilm formation	BES operation
Component	Amount	Amount
Yeast extract	2.00 g·L^{-1}	----
NH_4Cl	0.50 g·L^{-1}	0.50 g·L^{-1}
$MgSO_4 \cdot 7H_2O$	0.50 g·L^{-1}	0.50 g·L^{-1}
$CaCl_2 \cdot 7H_2O$	0.25 g·L^{-1}	0.25 g·L^{-1}
NaCl	2.25 g·L^{-1}	2.25 g·L^{-1}
$FeSO_4 \cdot 7H_2O$ solution	2.00 mL·L^{-1}	2.00 mL·L^{-1}
Trace element solution SL-10	1.00 mL·L^{-1}	1.00 mL·L^{-1}
Selenite-tungstate solution	1.00 mL·L^{-1}	1.00 mL·L^{-1}
K_2HPO_4	0.35 g·L^{-1}	0.35 g·L^{-1}
KH_2PO_4	0.23 g·L^{-1}	0.23 g·L^{-1}
$NaHCO_3$	4.00 g·L^{-1}	4.00 g·L^{-1}
Vitamin solution	10.00 mL·L^{-1}	10.00 mL·L^{-1}

Supplementary Table S7. Medium based on Aulenta *et al.*, 2012 and modification during biofilm formation and BES operation with *Desulfovibrio* sp.

	Biofilm formation	BES operation
Component	Amount	Amount
Na-D-Lactate	1.12 g·L^{-1}	----
K_2HPO_4	0.40 g·L^{-1}	0.40 g·L^{-1}
NH_4Cl	0.50 g·L^{-1}	0.50 g·L^{-1}
$CaCl_2 \cdot H_2O$	0.05 g·L^{-1}	0.05 g·L^{-1}
$MgCl_2 \cdot 6H_2O$	0.10 g·L^{-1}	0.10 g·L^{-1}
$NaHCO_3$	1.50 g·L^{-1}	1.50 g·L^{-1}
$FeSO_4 \cdot 7H_2O$	1.39 g·L^{-1}	----
Trace element solution	1 mL·L^{-1}	1 mL·L^{-1}
Vitamin solution	1 mL·L^{-1}	1 mL·L^{-1}

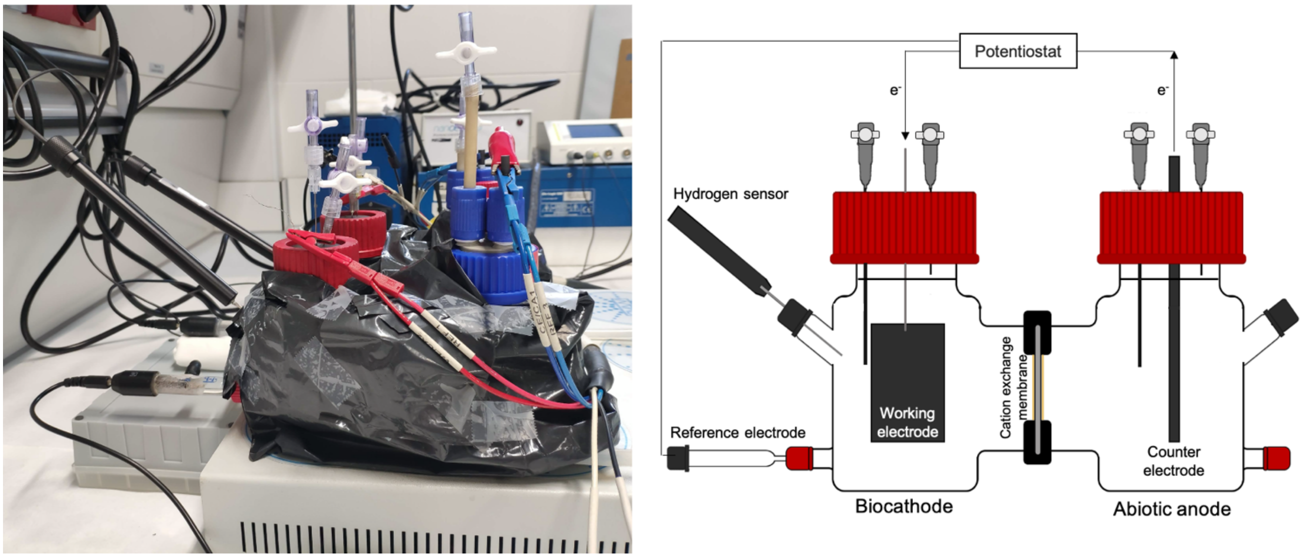

Supplementary Figure S1. Bioelectrochemical system (BES) configuration (left) and simplified scheme of the experimental setup (right). Line drawing on the righthand side was made from scratch using Adobe Illustrator v17.0 (https://adobe.com/). Picture is owned by authors.

References

1. Aulenta, F., Catapano, L., Snip, L., Villano, M. & Majone, M. Linking bacterial metabolism to graphite cathodes: electrochemical insights into the H_2-producing capability of *Desulfovibrio* sp. *ChemSusChem* **5**, 1080–5 (2012).
